# Alzheimer’s Research UK Research Conference 2026

**DOI:** 10.1177/23982128261454415

**Published:** 2026-06-11

**Authors:** Elizabeth M. Simzer, Jamie A. Elliott, Soraya Meftah, Sowmya Sekizar, Lauren F. P. Young, James H. Catterson

**Affiliations:** 1 Institute for Neuroscience and Cardiovascular Research, The University of Edinburgh, UK; 2 UK Dementia Research Institute, UK

**Keywords:** Alzheimer’s disease, dementia, Alzheimer’s Research UK

## Abstract

On 24–25 February 2026, Alzheimer’s Research UK held its annual research conference at Manchester Central Convention Complex and online. The meeting brought together over 700 researchers spanning molecular biology, data science, clinical trials, prevention, and patient engagement. Over 2 days of plenaries, parallel sessions, and discussions, a clear message emerged: the field of dementia research is entering a new, more hopeful era. This report summarises a meeting that highlighted how dementia research is moving beyond the search for a single solution and instead embracing a multidimensional, collaborative framework for precision care.

## Introduction

The Alzheimer’s Research UK (ARUK) Research Conference 2026 brought together researchers spanning basic, translational, and clinical disciplines. Opening with a powerful supporter address from Charlie Quirke, who has raised nearly £300,000 for dementia research in honour of his mother, actress Pauline Quirke, his remarks served as a moving reminder of the profound impact dementia has on families. This set an important tone for the meeting: that scientific progress must remain firmly grounded in patient experience.

## Clinical trials and therapeutic momentum

From molecular discovery to clinical trials and public involvement initiatives, the programme highlighted both the complexity of dementia and the breadth of expertise addressing it. This was reinforced in the opening plenary by Prof. Cath Mummery, who reflected on the rapid evolution of Alzheimer’s disease clinical trials, noting that “there is that feeling of acceleration, there is that positivity that we are changing things.”

The recent arrival of the first disease-modifying therapies targeting amyloid represents a historic milestone for dementia research (Sims et al., 2023; Van Dyck et al., 2023). However, speakers emphasised that this marks not an endpoint but the beginning of a more complex phase, in which additional therapeutic targets and combination approaches will be required. Alongside amyloid-β, increasing attention is being given to tau-directed strategies, including antisense oligonucleotides designed to reduce tau expression, reflecting a broader shift towards multi-target intervention (Shulman et al., 2026). As Mummery noted, the challenge now lies not only in developing additional therapies but also in determining how best to deliver them in real-world healthcare settings.

Across several sessions, discussions highlighted the rapid pace of therapeutic development alongside the need to refine clinical trial design to reflect the biological complexity of neurodegenerative disease. Future trials will increasingly rely on earlier intervention, improved patient stratification, and biomarker-guided enrolment. Incorporating patient perspectives was also highlighted as essential for improving accessibility and participation in trials. Together, these developments suggest that the next phase of dementia research will focus not only on discovering treatments but also on implementing them effectively and equitably.

## Biomarkers and the future of diagnosis

Alongside therapeutic advances, improving disease detection and monitoring remains essential for effective implementation. Rapid progress in fluid biomarkers is transforming dementia diagnosis and research. Several speakers highlighted the potential of blood-based biomarkers to enable earlier and more accessible diagnosis, reducing reliance on costly and invasive imaging such as positron emission tomography (PET) scans. These developments are particularly important as disease-modifying therapies enter clinical practice, increasing demand for scalable diagnostic tools.

Among the biomarkers attracting attention was plasma phospho-Tau217. In the closing keynote, Prof. Henrik Zetterberg noted that “pTau217 is essentially a marker of amyloid pathology.” While striking, this reflects a growing consensus that certain phosphorylated tau species may serve as reliable downstream indicators of amyloid-β accumulation (Bucci et al., 2025), helping to identify patients with Alzheimer’s earlier and to improve clinical trial selection.

However, speakers emphasised that biomarker interpretation remains complex. Data from Zetterberg showing that detected levels of blood-based biomarkers varied with storage temperature and time highlighted a need for greater standardisation in sample processing, particularly in multi-centre trials. Relying on single measurements risks oversimplifying neurodegenerative biology. Instead, discussions highlighted the importance of biomarker ratios and combinations to improve diagnostic specificity and account for co-pathology, which is increasingly recognised in neurodegenerative disease.

Integrating fluid biomarkers with genetics, imaging, and clinical phenotyping will therefore be key to enabling precision medicine in dementia research. By supporting earlier detection and patient stratification, these tools are beginning to reshape clinical trial design and the future of dementia diagnosis and care.

Importantly, the increasing sensitivity of diagnostic tools is revealing substantial biological diversity in neurodegenerative diseases. Rather than defining a single disease trajectory, biomarker profiles highlight variation in pathology, progression, and treatment response. As several speakers noted, advances in biomarker science are closely linked to efforts to understand disease heterogeneity and develop precision approaches.

## Heterogeneity and precision approaches

As diagnostic tools improve, they increasingly reveal the biological complexity of neurodegenerative disease. Heterogeneity in pathology, disease progression, and treatment response emerged as a recurring theme across the meeting, reinforcing the need to move beyond one-size-fits-all approaches towards more personalised interventions.

Advances in artificial intelligence and data science featured prominently in this effort. Several speakers presented new computational approaches for integrating large-scale datasets, including proteomics, genome-wide association studies, and polygenic risk scores. By combining these data types, researchers are beginning to identify shared molecular signatures across conditions such as Alzheimer’s disease, frontotemporal dementia, Parkinson’s disease, and amyotrophic lateral sclerosis. These approaches offer new opportunities to stratify patients, understand disease mechanisms, and ultimately guide more precise therapeutic strategies.

## Prevention in focus: Drugs, lifestyle, or both?

**Figure 1.**
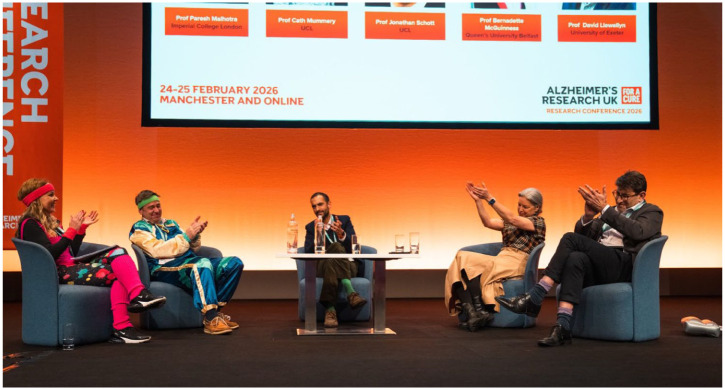
Debating dementia prevention proved to be something of a workout for the panel. From left to right: Professors Bernadette McGuinness, David Llewellyn, Paresh Malhotra, Cath Mummery, and Jonathan Schott. Photo credit: Rebecca Oliver.

While therapeutic innovation dominated many discussions, prevention remained an important focus. This was reflected in a lively debate chaired by Prof. Paresh Malhotra (Figure 1).

Professors Cath Mummery and Jonathan Schott argued that advances in disease-modifying therapies place pharmacological intervention at the centre of future progress. In contrast, brightly coloured shell suits and 1980s sweatbands briefly replaced typical conference attire as Professors David Llewellyn and Bernadette McGuinness championed lifestyle interventions such as exercise and cardiovascular health as powerful tools for reducing dementia risk.

Delivered with humour, the debate captured a central question for the field: whether progress will be driven primarily by therapeutics, prevention strategies, or a combination of both.

## Scientific highlights and emerging leaders

**Figure 2.**
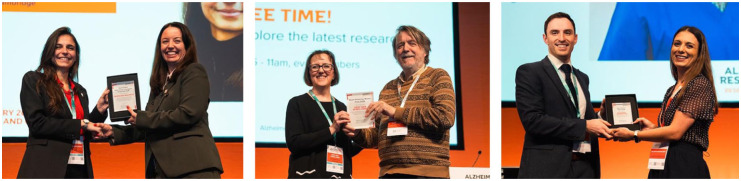
Prize winners at the Alzheimer’s Research UK Research Conference 2026. From left to right: Dr Maura Malpetti with Dr Sheona Scales, Prof. Tara Spires-Jones with Prof. Sir John Hardy, and Dr Robert McGeachan with Dr Leah Mursaleen. Photo credit: Rebecca Oliver.

Several prize lectures recognised outstanding contributions to dementia research across career stages, with a notable focus on tau biology and synaptic pathology (Figure 2).

The David Hague Early Career Investigator Prize was awarded to Dr Maura Malpetti for her work investigating neuroinflammation in frontotemporal dementia using PET imaging markers of tau and microglial activation (Malpetti et al., 2023). The Stuart Pickering-Brown lifetime achievement prize recognised Prof. Tara Spires-Jones for her dedication to dementia research and her significant contributions in mentoring the next generation of neuroscientists. Her research into the mechanisms of neurodegeneration continues to shape the development of new therapeutic strategies (Colom-Cadena et al., 2023).

The Jean Corsan Prize was awarded to Dr. Robert McGeachan for work showing that oligomeric tau can spread trans-synaptically in progressive supranuclear palsy using post-mortem tissue and live human brain slice cultures (McGeachan et al., 2025a). Notably, McGeachan completed his PhD in the Spires-Jones laboratory, highlighting two prize-winning contributions from the same research group. Similar slice culture approaches were also presented by Dr. Soraya Meftah, demonstrating their potential for mechanistic studies of Alzheimer’s disease and related dementias (McGeachan et al., 2025b).

## Posters and emerging themes

Nearly 250 posters highlighted the breadth of UK dementia research. Risk factors and biomarkers were among the most prominent themes, reflecting growing interest in blood-based and digital diagnostic approaches. Novel digital markers ranged from electroencephalogram (EEG) and sleep monitoring to keyboard-typing patterns, while risk factor studies suggested that early-life adversity may influence dementia risk decades later.

Artificial intelligence featured widely across posters compared to previous years, including one study applying large language models to analyse informant interviews to detect early cognitive impairment in people with suspected dementia (Kaur, 2026).

Mechanistic work remained equally prominent, with studies exploring neuroinflammation, *APOE* biology, mitochondrial function, lipid metabolism, and synaptic dysfunction. Collectively, these studies reflected a broader shift towards a more integrated view of dementia combining neuroimmune, metabolic, vascular, and circuit-level dysfunction.

## Community, collaboration, and career development

The conference also hosted workshops and career development sessions aimed at supporting researchers at different stages of their careers. Topics included patient and public involvement in funding applications, strategies for translating research into impact, and navigating barriers faced by early-career scientists.

A particularly well-attended fellows’ session on “Shaping Your Future as a Research Leader,” led by Prof. Charles Ffrench-Constant, Lydia Beaton, and Dr. Sarah Marzi, offered candid reflections on building a research career. Speakers highlighted the importance of maintaining momentum in early independent projects, seeking mentorship, and making strategic decisions around team building and funding. The discussion also emphasised the value of engaging with institutional research offices and donor networks to increase visibility and create new funding opportunities.

## Expanding access through digital engagement

The continued availability of an online format enabled broader participation from researchers unable to attend in person, including carers and those with limited childcare support.

Virtual attendees reported an engaging experience, with plenary lectures and panel discussions translating well online. Interactive tools such as Slido allowed remote participants to follow audience questions and contribute to discussions, although expanding online access to workshops and networking could further improve accessibility at future meetings.

## From momentum to implementation

In summary, the ARUK Research Conference 2026 reflected a field undergoing significant transition. Discussions highlighted a shift from questioning whether disease modification is possible to focusing on how emerging therapies can be implemented safely and effectively.

Several key questions emerged across sessions, including:

- How can fluid and imaging biomarkers be standardised and integrated for reliable diagnosis and monitoring?
- Which biological subtypes should guide patient selection and therapeutic decision-making?
- How can disease-modifying therapies be delivered safely and equitably at scale?
- How should prevention strategies complement pharmacological intervention?
- How can artificial intelligence and multi-omic data be translated into clinical practice?

Together, these challenges highlight a field moving beyond discovery towards implementation. The overall mood of the meeting reflected growing confidence among researchers and clinicians that the field is entering a new phase defined not only by scientific breakthroughs but also by their translation into meaningful benefits for people affected by dementia.
